# Metachronous testicular seminoma after radiotherapy and chemotherapy: a case report

**DOI:** 10.1186/s12957-016-0902-9

**Published:** 2016-05-16

**Authors:** Marcelo Di Gregorio, Marie Cécile Nollevaux, Francis Lorge, Lionel D’Hondt

**Affiliations:** Urology Department, CHU UCL Namur, 1 Av Gaston Thérasse, B-5530 Yvoir, Belgium; Pathology Department, CHU UCL Namur, Yvoir, Belgium; Oncology Department, CHU UCL Namur, Yvoir, Belgium

**Keywords:** Testis, Metachronous tumors, Seminoma, Radiotherapy, Chemotherapy

## Abstract

**Background:**

Bilateral testicular neoplasia is rare, with an incidence ranging from 1 to 5 %. Long-term survival has improved in recent years due to advanced diagnostic approaches and new therapeutic methods that are highly effective against germ cell tumors.

**Case presentation:**

We present the case of a patient with a primary seminomatous testicular tumor, who developed a contralateral metastasis and a subsequent metachronous tumor following chemotherapy and consolidation radiotherapy treatment.

**Conclusions:**

Strict follow-up, including physical examination and ultrasound examination of the contralateral testis, enabled early diagnosis of the second tumor, giving the patient a high likelihood of a definitive cure.

## Background

Testicular germ cell tumors (TGCTs) represent 1–2 % of all cancers in men and are the most common solid tumors found in male adolescents and young adults between 15 and 35 years of age [1]. The mean age at diagnosis is 34 years (median, 39.5 years) [2]. Most testicular tumors (95 %) arise from germ cells and can be divided into two main groups: seminomas and non-seminomas. Previous TGCT is a risk factor for contralateral malignancy development [3]. Compared with age-matched populations, patients with a history of TGCT show a 23–27 times greater relative risk of developing a contralateral germ cell tumor [4].

Bilateral testicular germ cell tumors (BTGCTs) occur with a global incidence ranging from 1 to 7.8 % [1, 5, 6]. Bilateral testicular tumors that occur simultaneously are termed synchronous tumors, while those occurring at different times are termed metachronous tumors. Metachronous BTGCTs are twice as prevalent as synchronous tumors [7] and are defined by an interval of > 6 months between neoplasm occurrences [8]. In cases of metachronous BTGCT, the second tumor usually occurs within 5 years after the first tumor.

## Case presentation

A 35-year-old man with no known risk factors for testicular malignancy presented at the urology department with a right testicular mass causing painful swelling. He had been experiencing discomfort and heaviness for 10 days. His general practitioner had started antibiotic and anti-inflammatory treatment a week prior to his arrival at our department. The patient had no past medical history of testicular issues.

**Fig. 1:**
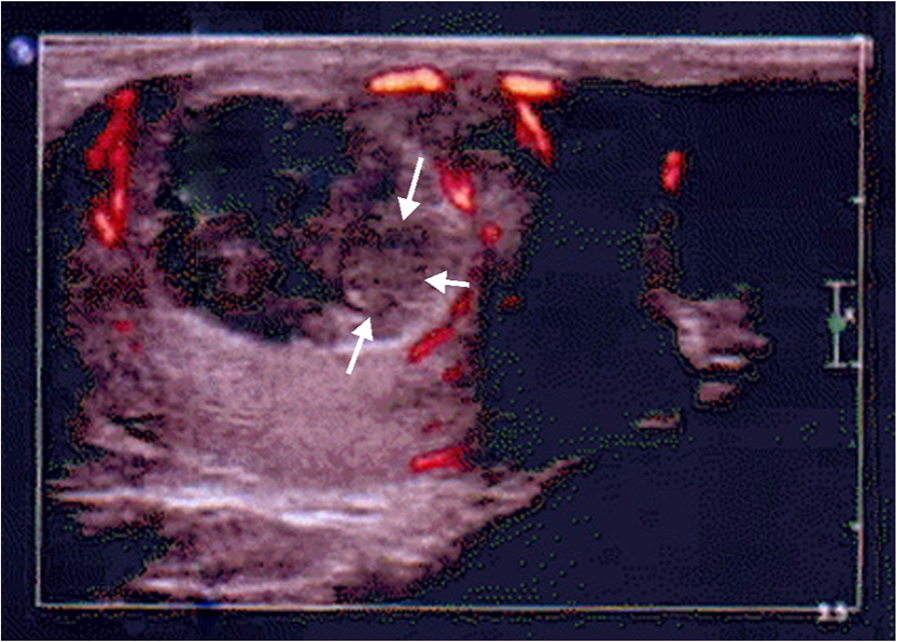
Testicular ultrasound showing an 18 mm × 12 mm × 25 mm heterogeneous hypoechogenic mass localized to the upper pole of the right testis (*arrows*)

Physical examination revealed a lump, which testicular ultrasound confirmed as an 18 mm × 12 mm × 25 mm heterogeneous hypoechogenic mass localized to the upper pole of the right testis (Fig. 1). A computed tomography (CT) scan showed no evidence of abdominopelvic or thoracic metastases. The blood serum tumor marker levels were as follows: human chorionic gonadotropin (HCG) < 1.20 U/ml (normal is < 5.01 U/ml); α-fetoprotein (AFP) = 3.4 ng/ml (normal is < 7 ng/ml); and lactate dehydrogenase (LDH) = 599 IU/l (normal is 313–618 IU/l).

A right inguinal radical orchiectomy was performed in September of 2009. Histological examination revealed a pure seminoma of 4 cm × 2.5 cm, without lymphatic, vascular, or tunica albuginea infiltration. The tumor node metastasis (TNM) classification was pT1pNxpMx according to the Union for International Cancer Control (UICC) staging system, seventh edition. Two weeks after the surgery, this case was discussed during a multidisciplinary uro-oncology meeting. From October 21 to November 10 of 2009, the patient underwent adjuvant radiotherapy with doses of 25.2 Gy delivered to the paraaortic lymph nodes in 14 fractions.

**Fig. 2:**
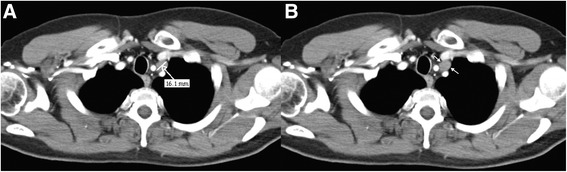
**a**, **b** Full-body computed tomography scan showing a 16-mm lymph node under the left collarbone (*arrows*)

**Fig. 3:**
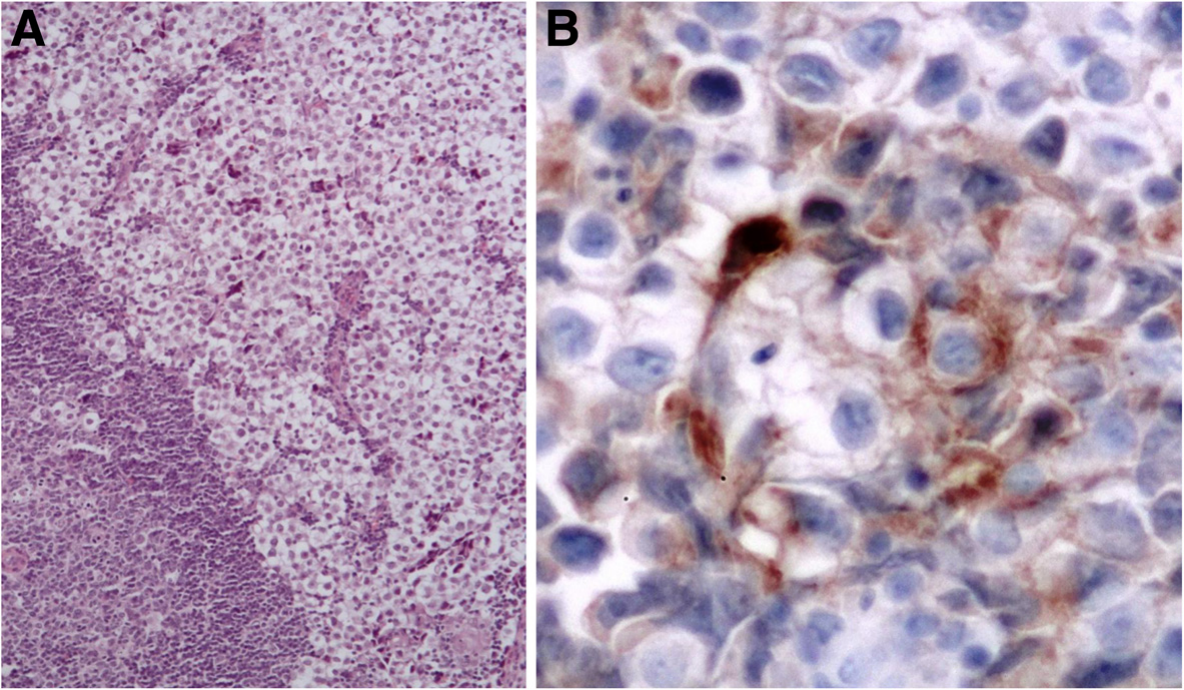
Histological examination revealed a typical seminoma. **a** Hematoxylin-eosin staining, ×200 magnification. **b** Immunoperoxidase staining using an anti-PLAP antibody, ×400 magnification

The patient was considered to be disease-free and received follow-up in accordance with our standard protocol, which includes chest and abdominal CT, physical examination, and tumor marker assessment every 4 months for the first 2 years, and testicular ultrasound of the contralateral side once each year. A total-body CT scan at 1 year after radical surgical treatment showed a 16-mm lymph node under the patient’s left collarbone (Fig. 2a, b). The lesion was confirmed by positron emission tomography (PET) scan, and surgical node excision was performed. Histological examination revealed a typical seminoma (Fig. 3a, b). Chemotherapy was initiated with a bleomycin, etoposide, and cisplatin (BEP) protocol administered every 21 days for 2 cycles from October to December of 2010.

**Fig. 4:**
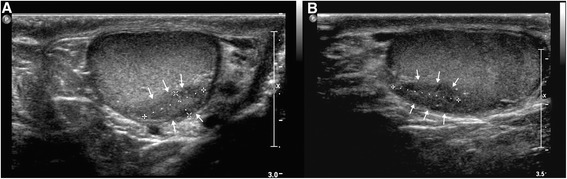
**a**, **b** Ultrasound of the left testis revealing a 15 mm × 6 mm node with microcalcifications (*arrows*)

Four years later, a follow-up ultrasound of the left testis revealed a 15 mm × 6 mm node with microcalcifications (Fig. 4a, b). Blood serum tumor markers were normal, and a CT scan showed no evidence of abdominopelvic or thoracic metastasis. The possibility of radical or partial orchiectomy was discussed with the patient. In March of 2014, the patient underwent left inguinal testicular exploration of the lesion with ultrasound image guidance and excisional biopsy. Analysis of frozen biopsy sections revealed a seminomatous tumor with an intense chronic granulomatous inflammatory lesion. Due to the diffuse nature of the tumor, radical left orchiectomy was performed. The final pathological diagnosis was a pure seminoma that presented as isolated and scattered neoplastic cells within an inflammatory and granulomatous reaction and multifocal intratubular germ cell neoplasia (IGGNU).

The patient remained under surveillance and received androgen replacement therapy with long-acting testosterone undecanoate every 12 weeks (Nebido®). A bilateral testicular prosthesis was proposed but was refused. Sperm cryopreservation was not performed because the patient had children and did not desire any additional offspring. Follow-up was performed following the standard protocol. At 2 years after left radical orchiectomy, the patient remained disease free. At the most recent visit, the patient reported maintenance of libido, no adverse effects from the androgen replacement therapy, and comfortable sexual activity and quality of life.

### Discussion

Reported TGCT incidence rates from multiple countries between 1991 and 2011 show geographical variations, with the highest rates observed in Denmark. Over recent decades, TGCT prevalence has gradually increased in most populations of European origin and in the USA [9, 10]. Some studies suggest an increased incidence of bilateral disease in the post-chemotherapy and radiotherapy era [11, 12]. A retrospective review shows a threefold higher incidence of bilateral testicular cancers in the post-chemotherapy era compared to the pre-chemotherapy era [13]. The apparent increase in the number of metachronous tumors may reflect the increased life expectancy of the general population as well as the prolonged survival associated with higher cure rates for initial tumors.

A systematic literature review—including 50,376 men with TGCT between 1991 and 2011 from many countries—reported a BTGCT prevalence of 1.82 % [7]. Among those with BTGCT, 69.2 % had metachronous tumors and 30.8 % had synchronous tumors. Several studies indicate that metachronous testicular tumors seem to be more frequent than synchronous ones [14, 15]. Bilateral metachronous TGCT was first described in a case report in 1942. Metachronous testicular cancer is diagnosed when at least 6 months elapse between the appearance of the first tumor and the second tumor and when there is an ultrasound-documented absence of a contralateral mass at diagnosis of the first tumor.

Among patients with metachronous tumors, the mean age at diagnosis of the first tumor is 28 years old and the mean age at diagnosis of the second tumor is 35 years old [11]. Our present patient was 36 years old when the first tumor was diagnosed and 40 years old when the second tumor was diagnosed. In 70 % of cases, the second testicular malignancy arises within 5 years after the first TGCT [2]. Seminoma is the most common histological type of bilateral testicular cancer, comprising approximately 68 % of such cases [9], as well as the most common histological type of metachronous tumor [16]. When the second tumor is a seminoma, the median interval between tumors seems to be longer (~10 years) [17]. There have been 25 reported cases of BTGCT in which the contralateral testicular tumor occurred 20 years or more after the original tumor. Within a series of 25 cases, 4 cases involved a second tumor that occurred at least 30 years after the original testicular tumor, with the longest interval being 40 years [11, 12]. Contralateral testicular seminoma can occur even at an advanced age, underscoring the importance of life-long follow-up for these patients [17, 18].

The incidence of metachronous germ cell tumors in patients diagnosed with a seminoma is influenced by the patient’s age at the time of the initial diagnosis. Evidence suggests that men who develop a seminoma when they are 30 years of age or younger may be at greater risk of developing a second tumor [2, 15]. Patients diagnosed with a seminomatous tumor at less than 30 years of age show an increased risk of relapse in the following 15 years compared to men who are over 30 years old at diagnosis (3.1 vs 1.2 %) [19].

Although the etiology of BTGCT remains unknown, both genetic and environmental causes are implicated. Presently known epidemiological risk factors for TGCT development include a history of cryptorchidism, Klinefelter syndrome, the presence of a contralateral tumor, infertility, and a history of testis cancer in first-degree relatives [17]. The elevated risk in family members and associations with inherited genotypes suggest genetic causes [20, 21]. On the other hand, testicular cancer incidence rates nearly doubled in industrialized countries between 1975 and 2007, suggesting an influence of environmental factors [9, 22]. Our present case involved no known genetic or environmental risk factors.

In our present case, serum markers were negative both at the diagnosis of the first tumor and at tumor recurrence. This is in accordance with the typical presentation of a seminoma. Most second tumors are discovered by the physician via scrotal ultrasonography or by the patient via testicular self-examination. Ultrasonography is a safe and simple screening procedure. One major difficulty regarding the diagnosis of second tumors is that patients may be reluctant to seek help due to fear of castration.

Ultrasound detection of microlithiasis in the contralateral testis is associated with a 30-fold increase in the risk of presenting with a second TGCT, and diagnosis of the first tumor is associated with a 5–8 % risk of testicular intraepithelial neoplasm (TIN) in the contralateral testis. These data highlight the need for long-term surveillance to support early detection of the second TGCT. Within 7 years, 70 % of all TINs will progress to invasive neoplasia [23, 24], although this risk is somewhat lower among patients who undergo chemotherapy for their first tumor. The 5-year survival rates for men with synchronous and metachronous bilateral testicular tumor are 88 and 95 %, respectively [7], suggesting that metachronous tumors have a more favorable survival outcome than synchronous tumors. Synchronous tumors are also associated with more advanced disease than metachronous tumors [7]. Among patients with bilateral testicular cancer, 70 % present with stage I disease upon diagnosis of the second tumor. This is most likely due to close follow-up and increased patient awareness.

The optimal management of patients with intratubular germ cell neoplasms remains controversial. The choices include surveillance and irradiation of the contralateral testis. Since radiotherapy can result in infertility and may affect Leydig cell function [1], surveillance is an important part of TGCT follow-up. Clear guidelines are also lacking for treatment of bilateral testicular tumors. Treatment of the second tumor is based on the stage and histology [11]. The incidence of contralateral testicular cancer is not significantly influenced by the use of radiation therapy for the initial testicular cancer [25].

Treatment for advanced germ cell tumors includes combination chemotherapy with bleomycin, cisplatin, and etoposide, followed by surgical salvage for residual disease. Depending of the patient’s risk profile, 3–4 cycles of chemotherapy are needed [26]. The patient in our present case received adjuvant radiation therapy after the onset of the first tumor as well as chemotherapy. Additionally, a metastatic lymph node was removed at relapse, which occurred long before the diagnosis of the second tumor. Notably, 5 years elapsed between diagnosis of the first and second tumors. A left radical orchidectomy was performed to eliminate the recurrent tumor due to its diffuse character and the history of metastases. Sparing the testis would have carried a risk of recurrence. Taking into account that he did not desire more children, the patient wanted radical surgery despite the need for hormonal replacement.

In the present case, the detection of a contralateral supradiaphragmatic lymph node 3 years prior to the contralateral testis diagnosis indicated metastatic relapse. A review by Cooper et al. reported that approximately 75 % of seminomas present as stage 1, with disease limited to the testis [27]. All tumors of germ cell origin have the propensity to metastasize via lymphatic pathways, which typically occurs in a sequential pattern, beginning with abdominal lymph node involvement, followed by successive involvement of lymph nodes in the chest and neck [28]. Wood et al. demonstrated that cervical metastasis is almost exclusively left-sided, with 21 of 23 patients showing disease in supraclavicular or scalene lymph nodes [28]. Metastatic tumors can also appear in locations outside of the direct line of spread from the primary site [29]. A review by Vledder reported that 4 % of seminoma patients showed cervical metastasis and that only 5 % of these patients had the neck mass as their initial disease sign [30]. Seminomas can metastasize to the supraclavicular lymph nodes, and tumors from the right testis can spread to the interaortocaval, precaval, and paraaortic regions, with crossover to left-sided lymph nodes. The left testis drains into the paraaortic and preaortic regions. Interaortocaval lymph node involvement occurs in higher-stage disease. From there, the tumors usually grow along the thoracic duct into the left supraclavicular lymph node and the subclavian vein and then show disseminated spread [31]. This hypothesis may be applicable to our present patient, since metastasis was not found elsewhere.

## Conclusions

Patients with a primary testicular tumor carry an increased risk of supraclavicular lymph node metastasis despite radiotherapy, and a metachronous second primary tumor can arise even with chemotherapy. A strict follow-up protocol involving physical and ultrasound evaluation of the contralateral testis supports early diagnosis of a second tumor, increasing the chance of a definitive cure. Notably, metachronous tumors may occur long after the initial tumor diagnosis. Treatment of the second tumor is dictated by its histology and its pathological and clinical stage at the time of diagnosis. Hormonal testosterone replacement can help promote a good quality of life.

### Consent

The patient gave written informed consent for the publication of this case report and any accompanying images. A copy of this written consent is available for review by the Editor-in-Chief of this journal.

## Abbreviations

AFP: α-fetoprotein
BEP: bleomycin, etoposide, cisplatin
BTGCT: bilateral testicular germ cell tumor
CT: computed tomography
HCG: human chorionic gonadotropin
LDH: lactate dehydrogenase
TGCT: testicular germ cell tumor
TIN: testicular intraepithelial neoplasia
TNM: tumor node metastasis
UICC: Union for International Cancer Control
